# Biographical Notices

**Published:** 1849-12

**Authors:** 


*Biographical Notices.*—In this number, our readers will perceive (we trust without disapprobation,) obituary notices of two distinguished members of the medical profession, lately deceased. We deem such notices not only appropriate as tributes of respect to the memory of the dead, and to the profession, of which they were honorable members, but calculated to interest, and at the same time to exert a salutary influence upon the mind of the medical reader. Entertaining this view, we shall continue the republication of similar articles, as occasions, unhappily, may require, and in our next number we shall present to our readers notices of the lives of the late Doctors George McLellan, of Philadelphia, John Harrison, of New Orleans, and Enock Hale, of Boston.

It is gratifying to see evidences of a disposition to perpetuate, in this, way, the lives of eminent brethren, and to secure from oblivion names which should not be lost to American Medical Biography.

